# A novel approach to expedite evidence to impact in pre-eclampsia: co-developed policy labs in Zambia and Sierra Leone

**DOI:** 10.1186/s44263-024-00116-8

**Published:** 2025-01-07

**Authors:** Katy Kuhrt, Chileshe Mabula-Bwalya, Harriet Boulding, Alice Beardmore-Gray, Alexandra Ridout, Osman Koroma, Betty Sam, Prince Tommy Williams, Francis Smart, Isabel Meleki, Meek Mwila, Mubanga Chileshe, Racheal Mawere, Alice Hurrell, Christabel Mbiiza, Cristina Fernandez-Turienzo, Jane Sandall, Bellington Vwalika, Andrew Shennan, Kate Bramham

**Affiliations:** 1 Department of Women and Children’s Health, King’s College London, London, UK; 2 University Teaching Hospital, Women and Newborn Hospital, Lusaka, Zambia; 3 The Policy Institute at King’s College London, London, UK; 4 Welbodi Partnership, Freetown, Sierra Leone; 5 Lifeline Nehemiah Projects, Freetown, Sierra Leone; 6 Department of Policy, Planning and Information, Ministry of Health and Sanitation, Freetown, Sierra Leone; 7 University of Zambia – King’s College London Maternal Health Collaboration, University of Zambia, Lusaka, Zambia; 8 Ministry of Health, Lusaka, Zambia; 9 Department of Obstetrics and Gynaecology, University of Zambia, Lusaka, Zambia

**Keywords:** Policy lab, Pre-eclampsia, Hypertensive disorders of pregnancy, Low-and middle-income settings

## Abstract

Pre-eclampsia is a leading cause of maternal and neonatal mortality; 30,000 pre-eclampsia-related maternal deaths occur annually, with 70% in Sub-Saharan Africa (SSA) and 16% in South Asia. We have shown that early, accurate detection of hypertension combined with planned early delivery in women with late preterm pre-eclampsia significantly reduces stillbirth and severe maternal hypertension. We describe co-development and delivery of policy labs, working with The Policy Institute (King’s College London), and local stakeholders in Sierra Leone and Zambia, to expedite integration of new knowledge into pre-eclampsia care pathways, to improve care for women and babies with the worst outcomes. Policy labs are a unique, user-centric engagement approach, bringing diverse stakeholders together in co-designing strategies for translation of evidence into policy and impact. Both labs were facilitated by local, well-respected female team members, and addressed co-developed questions: ‘How can we improve timely detection and appropriate action in women with pre-eclampsia?’ (Sierra Leone); ‘What are enablers and barriers to offering planned early delivery between 34 and 37 weeks?’(Zambia). Participants at both labs identified lack of pre-eclampsia awareness as a key barrier, and recommended local co-design of community-based strategies to increase access to timely pre-eclampsia management. We demonstrated  policy labs as an effective approach in two low-and middle income settings to facilitate transfer of new knowledge into policy and action.

**Supplementary Information:**

The online version contains supplementary material available at 10.1186/s44263-024-00116-8.

## Background

Pre-eclampsia, a leading cause of maternal and neonatal mortality, contributes to 30,000 maternal deaths and 500,000 neonatal deaths, the vast majority occurring in low- and middle-income settings (LMICs) [1–3]. Sierra Leone and Zambia have differing maternal mortality ratios (MMR) (443 [4] vs 213 per 100,000 live births), with pre-eclampsia related to 16% and 10% of deaths respectively [5]. Pre-eclampsia, diagnosed by the presence of new-onset hypertension after 20 weeks’ gestation with evidence of other organ involvement [6], is a complex multi-system disease related to abnormal placental development [7]. It can present during pregnancy, delivery or the postnatal period, with symptoms (headache, visual disturbance, abdominal pain, hand or face swelling), or may be asymptomatic. Pre-eclampsia can rapidly and unpredictably progress to severe complications, including eclamptic seizures, stroke, organ dysfunction, foetal growth restriction and stillbirth. Effective management involves early detection and control of raised blood pressure (BP), prevention of seizures and definitive management: delivery of the baby and placenta, which initiates disease resolution, but has to be carefully balanced against prematurity-associated risks to the baby, especially in LMICs with limited neonatal care [6, 8–11].

To improve prompt pre-eclampsia detection, often challenging in LMICs due to lack of accurate BP monitoring equipment and training [12], our team developed the CRADLE vital signs alert (VSA), an accurate, validated tool for BP and heart rate (HR) measurement in pregnant women, with an early-warning traffic-light system [13]. The CRADLE VSA intervention was associated with a 60% reduction in maternal mortality in Sierra Leone (RR 0.37 [95% CI 0.25 to 0.55], *p* < 0.0001) [14] (CRADLE-3). Our research in Zambia demonstrated that CRADLE VSA-based identification of hypertensive women followed by planned early delivery for late preterm pre-eclampsia was associated with a significant improvement in severe maternal hypertension and a 75% reduction in stillbirth (CRADLE-4) [15]. Expediting this new knowledge into policy and clinical practice is critical to improve maternal and neonatal outcomes. However, in Sierra Leone and Zambia, and globally, there are complex, multifactorial barriers preventing access to and uptake of care for pre-eclampsia. Thaddeus and Maine’s framework describes three delays contributing to poor obstetric outcomes, particularly in Sub-Saharan Africa (SSA) [16]: (1) decision to seek care; (2) arrival at a health facility; (3) provision of adequate care, each influenced by multiple actors and interacting obstacles, e.g. traditional and religious beliefs, patients’ and healthcare professionals’ knowledge, cost, distance, quality-of-care and resource availability. In this context, policy labs can provide a novel ‘user-centric’ engagement approach, through their unique emphasis on bringing diverse stakeholders from the target population together, dismantling hierarchies and combining various insights and experiences, in the co-design of strategies to bring emerging research evidence closer to policy and impact [17–19]. Hinrichs-Krapels et al. and The Policy Institute at King’s College London (KCL) developed the ‘Trust-Translation-Timing’ framework which advocates for coalition-building through the participation of diverse communities (‘trust’); work on the presentation of evidence to engage key stakeholders (‘translation’) and creating or leveraging ‘policy windows’ (‘timing’) [19]. In this article, we (a) describe the process of co-designing and delivering two pre-eclampsia-focused policy labs in Sierra Leone and Zambia as a novel approach to facilitate knowledge transfer, and (b) demonstrate their strengths and limitations as a strategy to expedite evidence to impact in LMICs.

### Reporting on our experience

Working with The Policy Institute, we applied Hinrichs-Krapels et al. and The Policy Institute’s (2020) eight-step process for delivering policy labs and the ‘trust-translation-timing’ model [19], to co-develop two policy labs with local partners in Sierra Leone and Zambia (Table 1).

**Table 1:** Implementing the 8-step policy lab process in two low- and middle-income settings based on the Trust, Translation, Timing Model [19]

	***Sierra Leone policy lab***	***Zambia policy lab***	***Both policy labs***
**Steps 1 and 2:** *Establishing the need and purpose of the policy lab* *(TIMING)*	• MMR 443/100 000 live births PE related to 16% deaths • Emerging evidence related to key aspect of PE management: early detection—*CRADLE-3* Trial: CRADLE VSA intervention associated with a 60% reduction in maternal mortality (RR 0.37 [95% CI 0.25 to 0.55], *p* < 0.0001). • **Timing:** *CRADLE-3* Evidence prompted MoHS-backed national CRADLE VSA scale-up, amidst reports of unprecedented improvements in MMR (1682/100,000 to 443/100,000 between 2000 and 2020)—drove appetite and motivation amongst policy makers for further progress.	• MMR 213/ 100,000 live births PE related to 10% deaths • Emerging evidence related to key aspect of PE management: planned early delivery (definitive PE treatment)—*CRADLE-4* Trial: Planned early delivery from 34 weeks of gestation in women with suspected PE associated with a 75% reduction in stillbirth. • **Timing:** *CRADLE-4* evidence well-aligned with Zambia’s recently launched 8th National Development Plan. Stakeholders keen to expedite into policy.	• PE remains a leading cause of maternal and fetal mortality and morbidity. • Challenges in accessing accurate BP monitoring equipment leads to delayed detection; uncertainty around timing of delivery leads to delayed or inappropriate management. • Multiple, complex barriers, influenced by diverse stakeholders, delay women accessing care (Three Delays Model)
**Step 3:** *Selection and invitation of participants* *(TRUST)*	• MoHS (policy lab collaborator) suggested participant list. Shared with policy lab facilitator and local community engagement specialist • Email and paper (hand delivered) invites issued by MoHs, followed up with SMS message reminders. • Invited Stakeholders: *Christian leader* *Muslim leader* *Traditional healer* *Traditional birth attendant* *Civil Society representatives* *Obstetricians* *Midwives* *Researchers* *NGO workers* *Policy-maker* *MoHS (govt) representatives* *Women (pre-recorded video).*	• University of Zambia (policy lab collaborator) curated participant list. • Email invites issued by University of Zambia • Invited Stakeholders: *Maternal health program officers* *Researchers* *Doctors* *Midwives* *Obstetricians* *Paediatricians* *Ministry of Health (government) representatives* *Women (pre-recorded video)*	Advised against inviting patient representatives due to concerns about their vulnerability in an unbalanced power dynamic; patients’ voices represented through pre-recorded videos by design.
**Step 4:** *Synthesize and communicate evidence* *(TRANSLATION)*	In addition to electronic version, participants received printed copy of briefing pack on arrival at policy lab.		• Policy briefing packs translating new evidence into an accessible format; co-developed by KCL, The Policy Institute, policy lab facilitators, and local stakeholders *(Additional file 1)* • Distributed to participants in advance of policy lab via email and What’s App.
**Step 5:** *Plan agenda and facilitation*	• Facilitator: Local Midwife with 30 years clinical experience; worked with MoHS; technical advisor to National CRADLE VSA scale-up in Sierra Leone • Agenda: included networking event post policy lab to enable cont’d discussion and planning of outputs in a relaxed, social setting.	Facilitator: Female doctor serving as District Health Director and maternal health advocate.	• Facilitators: involved in policy lab design & planning from outset. • Agenda: Break-out sessions interspersed with brief talks and video presentations, 1 day (10 am til 4 pm) (Additional file 2)
**Step 6:** *Conduct the policy lab*	• 7th March 2023, Freetown, Sierra Leone • 39 delegates • Ethnographic researcher carried out ethnographic observation of entire lab to capture intangible outputs.	• 14th February 2023, Lusaka, Zambia • 35 delegates	• Small, accessible conference venues selected in both cases • Group breakout Sessions: 1: Current attitudes & beliefs towards PE; 2: Barriers and facilitators to integration of new evidence; 3: Brainstorming interventions for change; 4: Feedback & discussion of selected interventions plus follow-up steps agreed. Each group assigned scribe to document outputs on flipchart. • Zambian delegates attended Sierra Leone policy lab and vice versa to facilitate cross-country learning
**Steps 7 and 8:** Report the results and create and support the new coalition	Technical working group formed amongst policy lab delegates to follow up on recommendations	Delegates completed a brief feedback survey to inform outputs and subsequent engagements.	• Flipchart notes transcribed and analysed thematically to identify key themes • See Tables 1 and 2 for summary of barriers contributing to delayed antenatal care access in Sierra Leone and Zambia respectively. • See Table 4 for theory of change summarising planned actions and anticipated outcomes. • Joint Policy Brief co-produced to promote fast follow-up and dissemination to delegates, colleagues and collaborators • In-depth lab reports prepared

*MMR* maternal mortality ratio, *PE* pre-eclampsia, *CRADLE-VSA* CRADLE-Vital Signs Alert, *RR* relative risk, *CI* confidence interval, *MoHS* Ministry of Health & Sanitation, *BP* blood pressure, *NGO* Non-governmental Organisation, *KCL* King’s College London

### Steps 1 and 2

#### Establishing the need and purpose of the policy lab

The primary purpose of these policy labs was to facilitate the accelerated achievement of national and global targets for the prevention of maternal and neonatal deaths through the integration of novel research evidence into clinical practice. CRADLE-3 trial showed the introduction of CRADLE VSA was most impactful in Sierra Leone, with reductions in maternal mortality and morbidity of 60 and 40% respectively [14], so the policy lab question was designed to facilitate the development of strategies to improve pre-eclampsia detection, ‘How can we improve timely detection and appropriate action in women with pre-eclampsia in Sierra Leone?’ The Zambia policy lab question, based on CRADLE-4 trial [15] focused on timely delivery; *What are enablers and barriers to offering planned early delivery between 34 to 37 weeks’ gestation, to women with pre-eclampsia?* Sierra Leone and Zambia were selected as settings for the two labs because they are the locations where CRADLE-3 and CRADLE-4 trials were undertaken, with populations most likely to benefit from the evidence. The two settings have similarities in challenges related to pre-eclampsia management but differ in their MMRs and resourcing. Delivering policy labs in both settings therefore increases the generalisability of our findings.

### Step 3

#### Selection and invitation of participants

In Sierra Leone, we co-designed the policy lab with our colleagues at the Department of Policy Planning and Information, and the Department of Reproductive Health and Family Planning, both within the Ministry of Health and Sanitation (MoHS). MoHS recommended a participant list which was shared and updated with the policy lab facilitator and a local community engagement specialist. Paper and electronic invitations were distributed by MoHS. We included a range of different stakeholders with diverse perspectives, for example Christian and Muslim religious leaders, traditional healers and healthcare workers. In Zambia, the primary delegate list, comprising Ministry of Health Officials, maternal health researchers, programme officers, obstetricians, midwives and paediatricians, all working across urban and rural settings, was curated by a team from the University of Zambia.

In both Sierra Leone and Zambia, building trust was a key priority, enabling honest, rigorous discussions, amongst a small group of diverse delegates, ranging from high-level government ministers, implementers who could identify barriers and facilitators to change in practice and community representatives. We were advised against inviting patients to attend the labs due to concerns about their vulnerability in an imbalanced power dynamic. To our knowledge, neither Zambia nor Sierra Leone have ‘professional patients’, nor community-based advocacy groups specifically targeted at pre-eclampsia, and as such patient voices and experiences were represented through pre-recorded videos.

### Step 4

#### Synthesise and communicate the evidence

Policy briefing packs for Sierra Leone and Zambia were developed collaboratively by KCL team members, policy lab facilitators and stakeholders in each country in an iterative process over a number of weeks (Additional File 1). We summarised emerging research evidence in the context of the existing literature, suitable for an audience with varying literacy levels, as a basis for discussion during the lab itself. Briefing packs were disseminated via WhatsApp (Sierra Leone) email, and participants received a printed copy on arrival.

### Step 5

#### Plan agenda and facilitation

In both settings, we gave careful consideration to the length of the policy labs (10 am–4 pm), with lunch and refreshments provided, and accommodation for those travelling from rural districts. Agendas were based on a structure provided by The Policy Institute and included a series of brief breakout sessions where groups were invited to discuss topic-based questions, interspersed with short talks and video presentations (Additional File 2). In Sierra Leone, a social networking event followed the lab, to enable continued discussion and evolution of ideas and follow-up plans. In Zambia, the policy lab was planned as part of a three-day meeting to disseminate CRADLE-4 trial results and plan future research collaborations. Local facilitators, familiar with the context and infrastructure of their respective settings, were identified to support development and delivery of both labs: in Sierra Leone, a midwife with over 30 years clinical experience, who worked with the Ministry of Health and Sanitation and was technical advisor to the recently completed national CRADLE VSA scale-up, and in Zambia, a female doctor who acted as a district health director and maternal health advocate. Facilitators were involved from the outset, including early discussions around tailoring policy lab questions, planning a focused agenda, moderating group debate at the group itself, and collating final outputs.

### Step 6

#### Conduct the policy lab

The policy labs were held in Lusaka, Zambia (14th February 2023) and Freetown, Sierra Leone (7th March, 2023). Both were held in conference venues carefully selected based on accessibility and suitability to small group breakout sessions and wider group discussions. In Sierra Leone, the policy lab was co-hosted by MoHS. This ensured good attendance (39 participants) with different stakeholder groups well represented, including civil society and grass roots organisation representatives, traditional healers, healthcare workers (tertiary, secondary and community level), national and local government representatives, NGO workers, Christian and Muslim religious leaders and researchers (from KCL, and Zambia to enable cross country learning). In Zambia 35 national and international delegates attended, including clinicians (obstetricians, midwives and neonatal care providers), researchers, programme officers from local and international maternal and child health-focused organisations based in Zambia and Ministry of Health officials.

Following an introductory presentation where the definition and aim of a policy lab approach were described, and key elements of the briefing pack summarised, pre-allocated, mixed breakout groups discussed current attitudes and beliefs towards pre-eclampsia (session 1); barriers and facilitators to integration of new evidence (session 2); brainstorming interventions for change (session 3); and feedback and discussion of selected interventions (session 4). Each group had a scribe who documented outputs on flipcharts. Groups fed back between breakout sessions and outputs were agreed in a final summary session. In Sierra Leone, a qualitative researcher carried out an ethnographic observation of the lab to capture the intangible outputs, e.g. the interpersonal dynamics. In Zambia, the final session consisted of a group discussion the about next steps and participants committed to championing specific initiatives.

### Steps 7 and 8

#### Report the results and create and support the new coalition

Flipchart notes and ethnographic field notes were separately transcribed and analysed thematically to identify key themes. Discussion points arising from sessions 1 and 2 highlighted factors contributing to delayed antenatal care access, cited as potential barriers to integration and uptake of novel research evidence in both settings (Tables 2 and 3).

**Table 2:** Factors identified by Sierra Leone lab participants contributing to delayed diagnosis and timely pre-eclampsia management

Delay	Examples
Misconceptions	Common misconceptions reported by lab participants held by some communities included beliefs such as: • ‘Fits are a sign that a woman is possessed by demons or devils’ • ‘Pre-eclampsia is associated with taking a bath at night’ • ‘Swollen feet show that the woman is expecting a male infant’
Transport costs and logistics	Distance to healthcare facility and cost of transport
Lack of trust in the quality of care received at the health facility or hospital	• Residual fear from Ebola of contracting disease at health facilities • Lack of trained staff • Many staff are volunteers with no regular salary • Poor adherence to guidance and management pathways • No accountability resulting in a lack of urgency to refer patients or deliver key interventions
Stockouts of basic equipment and medications	i.e. blood pressure machines, urine dipsticks, blood pressure medication, scanners
Cost of care	‘Free’ healthcare is not free; women are often asked to pay for consumables

**Table 3:** Potential barriers identified by policy lab participants in Zambia to planned early delivery

Barrier	Examples
Community concerns	Common beliefs and concerns held by some communities included: • Early delivery is a sign of infidelity • Fear amongst women of a ‘forced’, painful labour which may be bad for the baby. • Natural birth ‘prized’ by society: caesarean delivery = lazy, a failure. • Family’s ‘negative perceptions’ heavily influence decision making. Women’s voices often go unheard.
Widespread uncertainty of true gestational age amongst women and clinicians	Women often present to antenatal care late in pregnancy. Ultrasound scans are not widely available.
Fear of Neonatal Intensive Care Unit	Families: • Cost of care • Competing commitments—childcare, work, household Clinicians: • Planned early delivery would overwhelm NICU with sick babies. • Cost and logistical burden

*NICU* neonatal intensive care unit

The policy labs’ recommendations were broadly similar and focused on co-development of strategies to increase pre-eclampsia awareness, in order to facilitate acceptance/ uptake of novel management pathways amongst healthcare workers, women and key decision makers (partners, family members, communities) (Table 4). In Sierra Leone, participants prioritised the development of community-based, relatable approaches to dissolve misconceptions which prevent timely pre-eclampsia detection, as emphasised by a Christian Religious leader participant,

'Our role as religious leaders is to compliment health services. After today I will take this information away and preach it at to church services.'

**Table 4:** Key outputs summarised as a Theory of Change for policy labs held in Sierra Leone and Zambia

Inputs	Outputs		Outcomes–Impact	
	*Activities*	*Participation*	*Short-term*	*Long-term*
• Time • Money • Labour, i.e. Technical Working Group to support campaign delivery (including community engagement specialist; graphic designer; photographer) • Equipment and resources, i.e. anti-hypertensive medications; magnesium sulphate; antenatal corticosteroids	**1.Academic/ policy lab stakeholder: Co-production of Policy brief and Oral Presentations at International Conferences (e.g. NIHR Implementation Science Conference July 2023, UK; International Society of Hypertension Conference Sept 2023, India)** ***(Sierra Leone and Zambia)*** **2.Community based: Intentional community engagement through Pre-eclampsia health education campaign *(Sierra Leone and Zambia)*** -Co-develop a tailored approach with local stakeholders— e.g. co-development of a PE docu-film with pop-up cinema screenings to urban & rural communities; national media campaigns; development and distribution of educational infographics - Use ‘Agents of change’ to increase PE awareness amongst peers (‘nothing about us without us’) **3.Community Based: Birth preparedness and complication readiness** ***(Sierra Leone and Zambia)*** -Community education to promote early antenatal care attendance and raise awareness of obstetric danger signs -Maternal health promotion groups integrated into antenatal care **4.Hospital Based: Specialized centres for PE care** ***(Sierra Leone and Zambia)*** -Staff trained in care pathways integrating novel evidence -Mentoring and supervision, and refresher training to increase competence and accountability -Targeted PE resource management to prevent stock-outs (including integration of CRADLE VSA into National essential equipment lists) -Co-development of Shared Decision Making tools (infographic and animations) to increase knowledge and participation around i) early detection of PE; ii) Planned Early Delivery **5.Hospital Based: Staff engagement and education sessions** ***(Zambia)*** -Nationwide healthcare worker interviews regarding planned early delivery undertaken in collaboration with Zambia Association of Obstetricians & Gynaecologists & Ministry of Health -Engagement meetings to introduce planned early delivery evidence with midwives in all Lusaka delivery facilities -Promote and upscale Kangaroo Care	• Women with previous pre-eclampsia/ eclampsia (‘Change Agents’) • Education partners • Community leaders • Civil Society representatives • Religious leaders • Traditional healers • Traditional birth attendants • Teachers • Midwives • Nurses • Obstetricians • Paediatricians • Pharmacists • Hospital administrative and leadership staff • Researchers • Policy Makers • NGO partners • Government representatives	• Increased recognition amongst researchers/ policy makers of policy labs as an effective and feasible process to bring ‘evidence closer to policy’ in LMICs ***(International)*** • Raised PE awareness amongst multiple stakeholders nationally through: -Nema’s Choice docu-film screenings via pop-up cinema to > 1000 mixed stakeholders including women and communities - > 1500 PE infographics disseminated at markets and transport hubs -Multiple National radio and TV broadcasts ***(Sierra Leone)*** -National media campaign TV and radio broadcasts during week of World PE day **(Zambia)**	• Shared Decision Making as part of routine PE care pathways • ***(Sierra Leone and Zambia)*** • Universal access to accurate BP measurement & early PE detection • **(Sierra Leone *and* Zambia)** • Planned early delivery for suspected PE from 34 weeks integrated & observed as part of national PE guidelines • **(Sierra Leone *and* Zambia)** • Reduced maternal and neonatal mortality and morbidity related to Pre-eclampsia **(Sierra Leone** and** Zambia)**

*PE* pre-eclampsia, *CRADLE-VSA* CRADLE Vital Signs Alert, *NGO* non-governmental organisation, *LMICs* low- and middle-income countries, *BP* blood pressure

Healthcare workers amongst the participants identified the need for the development of Pre-eclampsia Care Pathways, with staff training and mentoring to improve accountability and adherence to guidelines in order to improve the quality of care and trust amongst women. In Zambia, participants recommended targeted education to hospital-based clinicians about planned early delivery, given anticipated resistance at hospital as well as community level. In Sierra Leone, the ethnographic observation captured interpersonal dynamics throughout the day. For example,

'There was a district health officer from Kailahun who knows the health system way too much and knows the lapses, and when she was talking…there was momentous laughter and clapping for hitting the nail on the head.' (Participant at policy lab Sierra Leone).

Both policy labs were timely. New research derived from CRADLE-3 [14] study followed by the recently completed MoHS backed national CRADLE VSA scale-up, in the spotlight of unprecedented improvements in Sierra Leone’s MMR (1682 in 2000 to 443 in 2020 [4], provided an opportune policy window, and an appetite to develop and support strategies to further accelerate progress towards SDG 3.1 [20]. This enthusiasm manifested in the immediate and self-directed formation of a technical working group amongst Sierra Leone policy lab participants to develop key recommendations. A joint policy brief compiled outputs for Sierra Leone and Zambia and presented an overview of next steps in an accessible format to promote fast follow-up, in addition to detailed individual policy lab reports. What’s App groups were set up for ongoing communication between policy lab attendees involved in delivering agreed outputs. The Zambia lab was held shortly after the launch of Zambia’s 8th National Development Plan (2022 to 2026) [21], and the announcement of the CRADLE-4 results [15], which stakeholders were keen to see rapidly translated into positive clinical impact.

Outputs were timed to coincide with World Pre-eclampsia Day (22nd May) which provided a global platform for dissemination beyond local participants and settings. In Sierra Leone, these included the co-creation of a docu-film about pre-eclampsia (‘Nema’s Choice’ https://www.youtube.com/watch?v=LUNTuDfptWM) with local film makers and actors, which was disseminated, along with specially designed infographics, to antenatal clinics, rural community hubs, universities and on social media. In both Sierra Leone and Zambia, radio and TV appearances promoted key messages about pre-eclampsia and described emerging research, and in Zambia, education sessions were held with midwives working at delivery facilities in and around Lusaka to consolidate new evidence into clinical care pathways.

#### Strengths of the policy lab approach

Both policy labs stimulated lively discussions amongst mixed stakeholders in small breakout groups (‘trust’), who discussed the research evidence, presented via a briefing pack, in the context of relevant barriers and facilitators, and developed interventions for change (‘translation’), which were disseminated on a receptive backdrop of positive progress and priority setting in maternal health at government and policy level in both settings (‘timing’).

In both Sierra Leone and Zambia, the policy lab approach was welcomed,

'The group was dynamic and you had these multi-disciplinary and diverse groups. There were interesting conversations generated. People were focused. They didn't want to leave their group discussions because there was a lot of interest.' (Facilitator, Sierra Leone)

Tangible outputs agreed at both policy labs materialised in the form of a Pre-eclampsia Awareness Campaign in Sierra Leone, substantial media engagement in both Sierra Leone and Zambia, and district-level meetings at health facilities conducting deliveries along with clinical guideline updates to reflect CRADLE-4 evidence in Zambia. Outputs also informed a successful funding application for co-development of tools to support Shared Decision Making for women with pre-eclampsia in Sierra Leone and Zambia.

#### Limitations of the policy lab approach

The women’s voices were represented via video link and, as a result, seemed quieter than the rest. Future labs should include *all* participants in person. More generally, the transition of a ‘successful workshop’ to a scalable tool for meaningful engagement with formal policymaking machinery has been widely identified as an ongoing challenge [19, 22–25]. Lewis highlights the fragility of policy labs, citing funding constraints, divergent reasoning styles and skill sets in knowledge translation and policymaking, with heavy reliance on political patronage [24]. In our case, whilst Ministry of Health officials attended both policy labs, there is currently no formal infrastructure through which recommendations can be actioned and integrated into policy. However, the policy lab can be seen as one part of a process towards bridging the ‘know-do’ gap. In both settings, the policy labs set a precedent, with manifestations of short-term change already apparent in the form of tangible, well-received community and hospital-based strategies.

#### Policy labs in the context of low- and middle-income settings

Compared to other knowledge translation strategies, from comprehensive theoretical frameworks [26], to more radical, creative methods being evaluated by teams in the UK, e.g. ‘MANIFEST’ [27], which considers whether artists or artistic approaches such as model making, poetry, wearables and illustrated stories can be useful in policymaking, the policy lab approach that we have described is pragmatic, cheap to deliver (Sierra Leone: £5328.00; Zambia £4644.00), whilst incorporating key principles that characterise ‘some of the best policy-making’ [27]: engaging a broad spectrum of people; going to where people are; using tailored modes of communication and engagement; and a dialogical approach. Policy labs have not yet been widely adopted in SSA. Olejniczak et al. deliberately excluded the small number of policy labs identified in SSA, as their research analysis aimed to include *nationally* grown policy labs, rather than international (e.g. UNESCO) labs hosted by a SSA country [22]. Kim et al. reviewed 133 Policy Innovation Labs, three of which were reported to have been held in SSA, but provided no further detail about their content or conduct [28]. Our policy labs in Sierra Leone and Zambia were well-received, and collaborative and highlighted a novel opportunity for expedited policy development, feasible in LMICs where the most vulnerable women, who would benefit most from novel interventions, are often the last to receive them.

## Conclusions

Policy labs are a novel tool to facilitate the transfer of new knowledge into policy and action. Our policy labs in Sierra Leone and Zambia successfully enabled the development of collaborations between academics, policymakers and community members (trust), synthesised new evidence into an accessible format (translation) and provided timely access to new knowledge (timing) to improve maternal and neonatal outcomes. Future policy labs should include the voice of the woman at the forefront of the discussion.

## Supplementary Information

Additional file 1. Policy lab Briefing Pack Sierra Leone

Additional file 2. Policy lab Agenda Sierra Leone

Additional file 3. Raw data: flip chart notes

Additional file 4. Raw data: ethnographic observation notes

## Data Availability

The datasets used and/or analysed during the current study are available in Additional Files 3 and 4 submitted with this manuscript.

## Abbreviations

PE: Pre-eclampsia
SSA: Sub-Saharan Africa
VSA: Vital Signs Alert
BP: Blood pressure
HR: Heart rate
MoHS: Ministry of Health and Sanitation
LMICs: Low- and middle-income countries
