# Supplementary material for: A novel approach to expedite evidence to impact in pre-eclampsia: co-developed policy labs in Zambia and Sierra Leone
Source: BMC Glob Public Health. 2025 Jan 7;3:3. doi: 10.1186/s44263-024-00116-8 (PMC11707905; doi:10.1186/s44263-024-00116-8)
Supplement: Supplementary file 2 — Additional file 2. Policy lab Agenda Sierra Leone [file 44263_2024_116_MOESM2_ESM.docx]

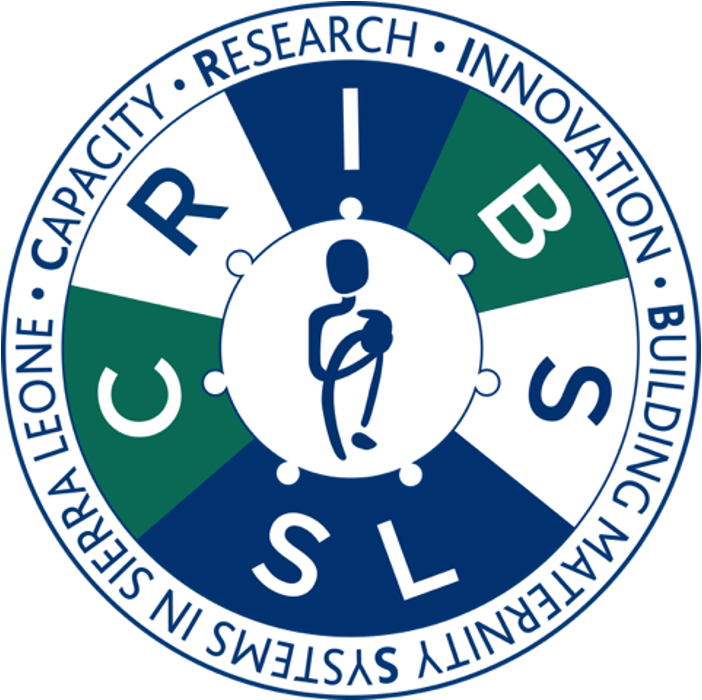

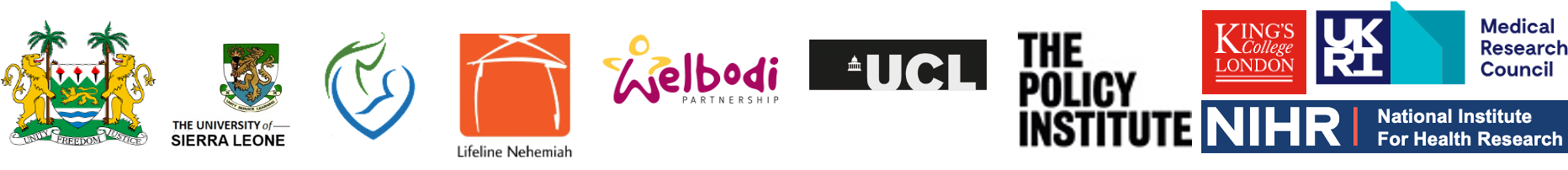

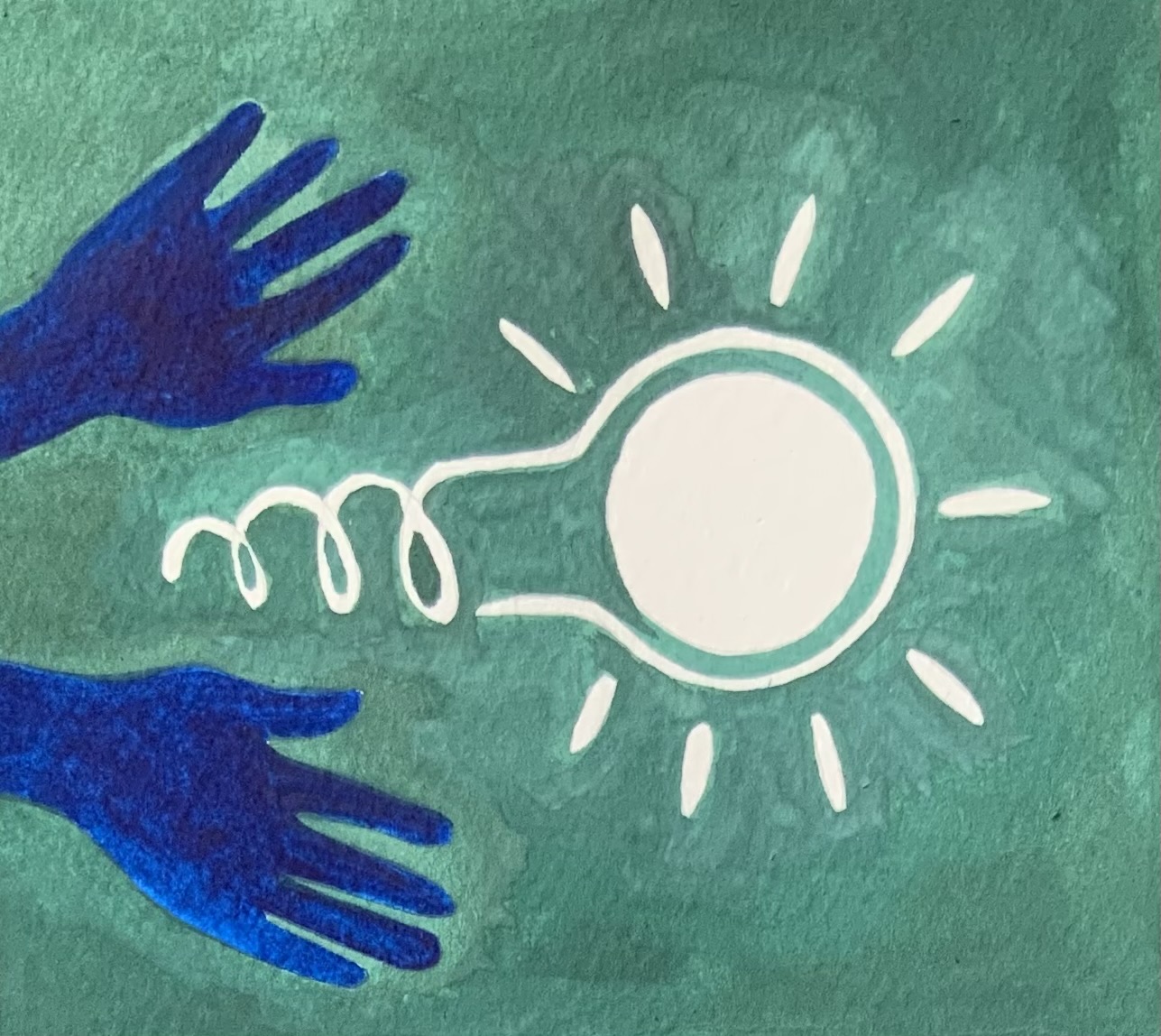


**POLICY LAB**

*How can we improve timely detection & appropriate action in women with*

*pre-eclampsia?*

7th March 2023

[@policyatkings](https://eur03.safelinks.protection.outlook.com/?url=https%3A%2F%2Ftwitter.com%2Fpolicyatkings&data=05%7C01%7Charriet.boulding%40kcl.ac.uk%7C71d6be006a7846fc384408dade7bfc9d%7C8370cf1416f34c16b83c724071654356%7C0%7C0%7C638066922193785874%7CUnknown%7CTWFpbGZsb3d8eyJWIjoiMC4wLjAwMDAiLCJQIjoiV2luMzIiLCJBTiI6Ik1haWwiLCJXVCI6Mn0%3D%7C3000%7C%7C%7C&sdata=d6XixYQXYirQkzvNHfUes%2FAE1TDx2akV%2F%2FMnz9rB%2F7A%3D&reserved=0)

@OBSevidence


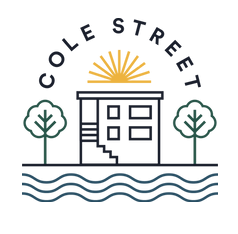

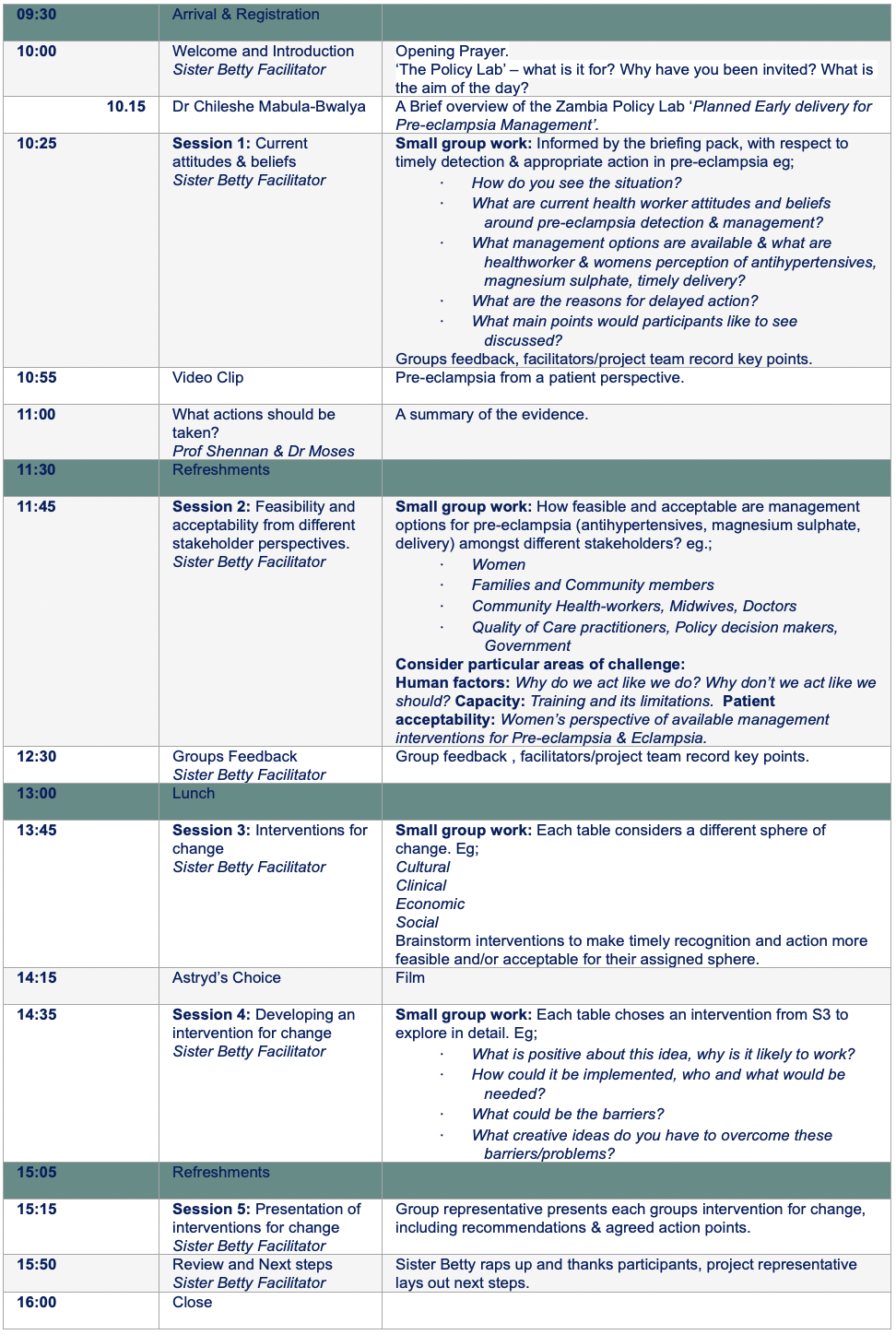


Join us afterwards

Join us afterwards at Cole street Guesthouse, Boutique hotel & Restaurant for light bites, fresh juice, tea, coffee & Live Music (performed by Reggie Thompson), **4.30pm to 6.30pm, 5 Cole Street, Freetown.**

*Cole Street, built by the late Lati Hyde-Forster, the first woman to graduate from Fourth Bay College and first African woman school principal in Sierra Leone remains committed to female empowerment, and champions education & vocational training for their mostly local female staff.*

*The kitchen presents the flavour of Sierra Leone using classic international techniques and fresh, organic, locally sourced, home-grown produce.*


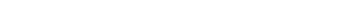

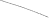

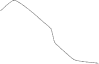

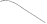

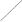

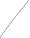

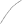

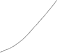

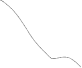

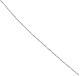

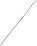

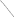

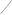

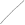

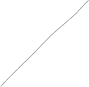

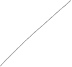

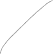

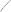

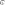

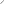

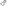

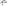

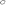

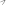

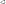

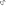

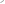

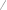

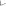

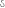

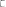

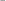

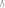

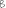

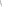

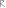

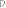

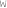

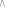

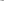

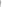

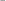

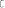

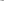

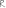

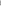

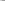

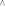

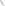

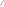

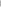

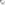

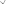

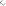

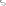

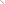

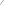

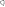

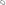

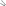

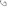

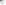

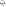

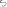

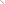

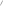

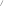

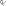

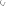

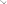

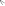

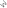


AIM of the Policy Lab

*To bring together researchers, clinicians, government representatives & community members to consider how evidence generated through recent research about timely detection & appropriate management of pre-eclampsia can translate into policies which help to reduce of maternal morbidity and mortality.*
