# Supplementary material for: A novel approach to expedite evidence to impact in pre-eclampsia: co-developed policy labs in Zambia and Sierra Leone
Source: BMC Glob Public Health. 2025 Jan 7;3:3. doi: 10.1186/s44263-024-00116-8 (PMC11707905; doi:10.1186/s44263-024-00116-8)
Supplement: Supplementary file 3 — Additional file 3. Raw data: flip chart notes [file 44263_2024_116_MOESM3_ESM.pdf]

Sierra Leone Policy Lab

SESSION ONE: CURRENT ATTITUDES / BELIEFS

Group -1

The Importance of Pre-eclampsia

Does everyone understand the dangers of Pre-eclampsia and what needs to do.

- A cultural belief is a challenge a barrier. All workers understand the dangers of Pre-eclampsia.

  As a result, most PHUs have a blood pressure machine. Mag-surf is always available.

  Healthcare workers have a protocol they always follow
- But there is a disconnect. There is a protocol but it is always being follow.

↘ Education & training (Gap) Because of lack of education & training, HCWs will go with the traditional beliefs in that community (especially in Rural Areas.

Mentoring & Supervision is recommended for HCWs to follow protocol in the management of Pre-eclampsia.

Attitudes

Traditional belief → delayed Action.

Group -2

- **Traditional beliefs**

  ↪ Traditional background trained to help manage.

  - Presence of TBA in hospital

- **Confusion around “what is Pre-eclampsia?”**
    - PET ↑ BP 20/40.
    - Presents swelling, headache
    - If ↑ BP continues-can develop fits.
    - Underlying cause___ placenta
    - Definitive treatment delivery baby and placenta.

- **Community beliefs PET.**
    - Symptoms PET= male infant
    - Fits = possessed; demons/devils.
    - Thought to be associated with certain foods i.e eggs.
    - Taking bath outside at night associated with PET

- **Solutions/improvements**
    - Education.
    - Consider radio programmes to public knowledge.
    - Ensure that, BP measured at health facilities.
    - Ensure prompt referral for conditions.

- **Barriers**
    - Community people have had previous negative experiences.
    - Poor health seeking behaviours
    - Present when very unwell.

- **Healthcare workers.**
    - Lack of knowledge /understanding
    - Acting upon signs/symptoms and BP

- Access to key diagnostics i.e dipstick.
- Poor compliance to guidelines/SOPs
- Lack of staff.
- Volunteers who are not remunerated.

- **Guidelines/SOPs**
  - Severe PET before viability people more open to terminate.
  - At gestational age decision to deliver more challenging.

⇒ These decisions/conversations impacted by patient's ideas, expectations and prior knowledge; but also the knowledge of the people caring for the patients.

- **Management**
  - MgSO4 –Issues with administration i.e loading dose not complete.
  - Confidence of providers low.
  - Especially primary levels ⇒ keen to transfer patients out.
  - Lack of familiarity.

- **Access to care**
  - Distance & coverage

Group -3

- Community: Negative outcomes in pregnancy have a demonic axiology.
- BP + Prokinoria

Use BP machine but don't necessarily relate BP reading to an action/urgency.

Many may not be taking accurate BP Readings.

KNOWLEDGE AMONGST HEALTHCARE WORKERS ⇒ MAY HINDER APPROPRIATE ACTION/MGMT

Readiness in facilities for PE is not there people may stay hours w/o referral.

- BP machines & other basics not available.

PE No.2 Killer for Past six (6) years, no change!

PHu managed by lower cadres ⟹ knowledge gap.

⟹ People don't want to work in many areas.

LACK OF ENABUNG ENVIRONMENT

Establishment of infrastructure should be comprehensive (Hospital with no housing is a non-starters.

WE NEED TO HAVE A STANDARD

POLITICAL WILL

= COMMITMENT & AUTHORITY

AVAILABILITY OF BASICS

ACCOUNTABILITY SYSTEMS NEED TO BE STRENGTHENED

- Health workforce + infrastructure + Procurement.

❖ KNOWING WHEN TO REFER

❖ Knowing what's beyond you.

ACCOUNTABILITY

- Knowledge GAP
- Lack of Adherence to guidelines
- No supervision.

AUDIT

ECLAMPSIA COMMON IN PARTICULAR GROUPS (eg teens)

ARE THEY BEING APPROPRIATELY TARGETE /IDENTIFIED /MANAGED?

Individualized and Respectful care dignity & compassion no matter what

(Poor, young, otherwise disadvantaged are both vulnerable & disenfranchised.

EFFECTIVE ANC

CERTIFYING EQUIPMENT QUALITY

Group-4

WHY DO MIDWNES/ CHO'S DELAY REFERRAL

- **PERCEPTION**
  "IF YOU REFER, YOU DON'T KNOW ANYTHING"

IMPLEMENTATION RESEARCH & BENCHMAKING

PATIENTS RIGHTS & CHARTER

Individual Monitoring

EXPERIENCE OF CARE ASSESSMENT

MDAs

Collaboration between line Ministries

TRANSPARENCY ABOUT DRUG STOCKS COMMUNICATION BT STORE KEEPER & IN CHARGE

SUB STANDARD BP MACHINES & OTHER EQUIPMENT

WELL-STOCKED EMERGENCY TROLLEY/CUPBOARD

- **DRUGS FOR EACH PATIENT FOR 48 HOURS**

IMPROVE DOCUMENTATION

MEDICAL SUPERINTENDENT + MATRON ACCOUNTABLE FOR THE PE CASE Moving nurses up & down.

PROCESS MAP FOR MGMT OF PE CASES

START PE PROTOCOL

ASSIGN "SPECIALIST" PE NURSES

Follow up on post discharges

PHu ⟶ Refusal to next level

CHc ⟶ Refusal to District Hospital.

District Hospital ⟶ Dx and commence tx

PRIORITISE PE

PCMH

1. CMO,/DIRECTOR RCH /RHFP & QOL Programme
   CLINICAL
2. DIRECTOR HR (Posting Midwives).

SITUATIONS

- **ACCESS:** Geographic barriers, not all case is available at PHUs.
- **ATTITUDES:** "Home treatment", PHu corruption reduces trust on health facilities; holly that, outcomes will be make it seeking treatment at PHu instead of from traditional healers;

lack of respectful , dignified & confidential cases during EBOLA; the public got carried to attend all out of fear of disease contraction; lack of respectful case.

- **DIAGONOSTICS:** Available CVSA for monitoring, maintenance & sustainability remains a challenge gaps in preventative maintenance, challenging ~~will~~ charging full shortages/ electricity occur.

- Quality of Case/Clinical Knowledge: Gaps in EMONC occur to midstate.

Reason for Delayed Actions:

- Clinical knowledge on EMONC

GROUP -5

- Going out to community to bring women in easily for prompts identification.
- Still many women delivering at home.
- Free in hospital, have to pay for home delivery.
- Hospital workers receive threats from people conducting home delivery.
- Knowledge gap in communities/amongst women-knowledge & advocacy needs↑.
- First delay-decision to go made by mother-In-Law/Husband.
- Need to focus on Sensitization ↑ & education.
- Some people in community are aware of PE, but from experiential learning, not from formal/structured sensitization/education.

- MSG-Maternity support groups, → promote early ANC & advise are symptom of PE, but need to ↑ focus on PE.
- Mg sulf & methyldopa available, no labetalol & hydralazine – poor compliance of (PCMH) HCW to SOP
- Eclampsia →stabilized & delivered no question.
- If severe PE –stabilize & discharge& admit-but many other admission due to other commitments-housework/kids.
- Women referee to PCMH-don't go ↓ trust in HCW (esp. after Ebola) need to think, why are women afraid of PCMH? Financial barrier stops people going.

SESSION II: FEASIBILITY/ACCEPTABILITY-for the Women

Group -1

1. Human Factors

- Lack of SOPs OR Non-adherence or lack of utilization (Tools).
- Knowledge gap in terms of the nutrients.
- Frequent rotation of trained staff
- Unclear role & responsibilities of service providers.
- Job description not specified in career pathways.
- Leadership and management issues.
- Recognition of the urgency & preparedness.

2. Capacity

- Training curricula to integrate relevant topics Pre-service.
- Practitioners no up to date with current trends.
- Lack of mentorship & Refresher trainings.
- Weak foundation of trainees.
- Inadequate supervision and monitoring.

- In adequate equipment drugs/medical supplies and other relevant resources.
- Funding limitation

3. Patient Acceptability

- Lack of knowledge of the disease condition & services available.
- Cultural interpretation of the condition & religious beliefs.
- Fear of poor out comes experience in health facility
- Negative attitude of Health workers, or poor Reception of HCWs (= Bad experiences) of women/patient:

Group -2

Women support groups-monthly meeting. One woman brings ten (10) pregnant women, educational meetings-danger signs, don't talk about death-should this change?

Trainers

Respectful maternity core

Culturally acceptable modifications delivery options

Partners involve in care

CHw focus on education + promote early ANC

Financial barriers-need to explain case should be free keynote, discussed at Sunday meetings.

Education

Include partner, people living with them, pastors /imams /chiefs/councilors.

Educate about risks to baby.

CHw role – raise awareness + refer –frequently women don't go, + it referred from CHs, women don't go.

- Training every 3 years.
- Selected from within community.
- Report to CHCs/PHVs.
- Outreach Clinics.
- Paid 150pm, quarterly pay
- CHW-identifies lead mother for women/mothers support group.

Need more feedback between CHw + CHc/PHV. + both reinforce messages.

Group-3

HOW FEASIBLE & ACCEPTABLE ARE MGMT OPTIONS FOR PE (MgS904, Antihypertensive, and Delivery) amongst different stakeholders.

QUALITY OF CARE PRACTIONERS PROGRAMME MGRS & POLICY DECISION MAKERS.

Antihtns: CCBS readily available.

Hydeadozine appeals to be reserved for District Hospitals.

FEASIBILITY IS WIDELY ACCEPTED

But training is needed (job-on training is needed (job-on training).

MIDWIVES have the skills but doctors don't want to be trained by them.

ECLAMPSIA **-** Doctors lack humility in training from others'

THIS CAN TRANSLATE INTO AN NABILITY TO TAKE ADVICE FROM JUNIOR OR OTHER CADRES AS A POLICY MAKER/PROGRAMME MGR.

Lack of adherence to:

- EMONC guidelines for use of PE interventions at operational level.
- Lack of accountability Systems.
- People are not adding lignocaine

  MgS04 → When people are reluctant to continue administering

- Lack of Communication effective education to optimize delivery of interventions.

  Are programme officers/policy people communicating with patents and frontline HCWs?

POLICY MAKERS ARE NOT ADEQUATELY TRANSLATING

- Policy
- Knowledge
- Patient Rights & Responsibilities

Nor enforcing accountability measures.
(2nd don't wait for a maternal death).

CHART
AUDIT

WHY IS NOT THE RIGHT THING HAPPENING?

→
- NO ENABLING
- ENVIRONMENT
- INFRASTRUCTURE DEVELOPMENT DOES NOT SUPPORT ACTUAL CLINICAL NEEDS

ISCONNECT
WEEN HIGH-LEVEL
SION MAKERS &
T-LNE DELIVERY

No spaces for relatives, no room for feedback or p communication.

No adequate iec TO PTS.

TIE CPD POINTS TO REGISTRATION

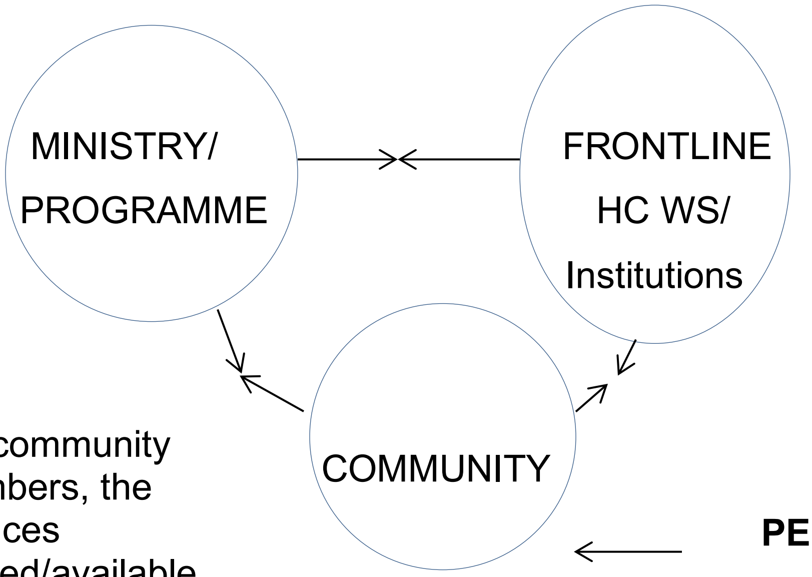

HEALTH TALK
MINISTRY AS A
BRIDGE
BETWEEN BTN
& CLINICIANS

For community members, the services offered/available are often below poor, but this way not be reaching technocrats.

PERCEPTION

ABANDONMENT,
NEGLECT, ABUSE
Treated as create

BIRTH WAITING HOMES

Did this help?

- No room for relatives to escort/support.
- Chiefs/communities were supposed to support food for mothers/TBAs (Re-purposed as BWHAs).

ACCOUNTABILITY

**IMPROVED DOCUMENTATION (HALTING)**
**SCORE CARDS**
**CHAMPIONS**

ADVOCACY/
REPRESENTATION

QoC - Communities need to be

Empowered to demand their rights

DECENTRALIZATION to community driver

DISCONNECTION FROM COMMUNITIES

THE FREE HEALTH CARE INITIATIVE

- SLESHI

  Meant to support but can be improved upon.

Group -4

- Quality of care
- Lack of investigation into adverse events
- Not acting on BP
- Lack of knowledge
- Explanation to patients lacking.
- Lack of compassionate care.

- Leads to disengagement of patient to access care
  - Need to pay for care and lack of resources.

- Financial bias.
  - Access to care –distance travel.
  - Lack of confidence in healthcare.
    - Women who have gone to hospital died.
    - Hospital = death sentence.
    - Avoid attending hospital.

- Social behavioral change health workers
- Lack of confidentiality.
- Lack of privacy/dignity when in hospital.
- Regular training /workshops

→ Refresher

- Follow ups in community.
- Lack knowledge /education of women.

→ Radio programme, teaching

- Empower/educate the patients.
    - They understand.
    - Can spread the message to their communities.
- Women believe in the healthcare provider of treatment.

→ Especially with clear explanation and teaching

- Delays in treatment may be due to financial concerns or need to obtain consent.

- EARLY delivery
    - If for benefit to the mother.
    - Need for clear understanding.
    - Concerns about infant survival

→ Especially previous pregnancy loss previous subfertility

- Women who are critically unwell may not have capacity to consent.
- Gender norms.
    - Involvement partner/father baby
    - Can delay care.
    - Can intervene care.

- Religious factors
    - Some religions termination forbidden.

- Perception full term is best.
- Women's expectations of care
    - May have to wait long times to receive care → delayed decision making, procedural delays.

- Traditional beliefs/concerns.
    - Exchanging babies in hospital
    - Baby theft.

- Women can be charged for services that are meant to be free.
- (+) international day

  Prematurity –community engagement.
- Special groups: adolescents
    - More vulnerable.
    - ↑ Risk all pregnancy complications.
    - Pre-pregnancy.

    → Schools: contraception, reproductive health, pregnancy.
- School health programmes.
- CSE

  Comprehensive

  Sexuality

  Education.

  → Community outreach.

Group -5:

Families + Community members.

- Stability & sacrifice
- Eclampsia = devile /demom curse
- Barriers to health care: trust in PHu corruption, expectual not ………….
- PHu still don't explain the disease or slept of management to Patients.
- Poor public awareness of PET & many ………………

- We need to move from ‘Sensitization’ to ‘intentional community engagement’.

1. 60% not educated → health information systems need to be improved.
   - Political divisions & dynamics can be barriers to patients seeking care at PHus→ patients don’t want to see PHu staff of opposition parties.
   - Communities rely on info from key stakeholders (ie leaders etc).

   → Awareness (up in rural) on national policies made in Freetown is not spread to rural women ………….

2. Rural leaders can increase public health awareness/education.
   - Localizing the word Eclampsia & explanations on case management
   - Traditional Myth contribution to first delays.
   - Hardly any knowledge on the disease & its management
   - Ru leaders are managing eclampsia, cases management

3. Stakeholders should be on the frontline of public health education & currently have insufficient awareness on PET. Awareness on policies don’t Awareness (up in rural pop) on national policies made in Freetown is not spread to rural.

4. CHWs should be sensitized on PET & Management.

5. Bi-Law Enforcement

TBA- ‘bad water’→ TBAs were paid to bring staff to PHU how stopped & back to own ways of many/providing services the traditional.

6. Respectful Maternity Care Training at Schools & Disciplinary Action for unethical conduct.

SESSION –III & IV

CLINICAL & HEALTHCARE INTERVENTIONS

Group -1

TRINING & EDUCATION

- Identification For Quality Service Delivery
- Awareness & Sensitization on Pre-Eclampsia & Eclampsia.
- Emergency & Response Preparedness
- Resources (Logistics)
- Ammonization of Sop for Timely & Better Intervention.
- Mentorship & Supportive Supervision.
- Establishment of Designated Unit for Pre-Eclampsia.

❖ **TREATMENT & MANAGEMENT**

↓

1. Establishment of a Unit for the management of Pre-eclampsia (Centre for Pre-eclampsia Care: CPC)

↓

2. Training and Capacity Building (Specialize Training of Personnel)___ Nurses
CHOs
Doctors

Etc

3. Resources for the Management of the Unit
   i. Basic Medical equipments
   ii. Human Resource
   iii. Drugs & Medical consumables
   IPC

4. Development of Policies, protocols and Guidelines.
5. Regular supportive supervision & mentorship for quality care.
6. Clinical Auditing Evaluation & Research.
7. Advocacy drive for sustained quality outcomes.

Group -2

Interventions for timely recognition and action

- ❖ **Cultural & Social**

- Patient education
  - Clear, simple, thorough
  - Different languages
- Peer to peer education
- Include Partner, family.
- Religious team involvement
  - Educate these community members.
  - They can guide women.
- Integrate PET/health seeking AN care into existing programmes i.e CRS.
- Traditional beliefs.
  - Challenging existing beliefs/counseling
- Preconception counseling
- Reproductive education for young women.

→ Contraception, pregnancy, pregnancy complications. (PIH, PET, eclampsia).

- Women empowerment in communities.
  → Involving males.
  → Awareness Rights.
- Public health messages.
  - Rural areas-town criers.
  - Radio-local languages.
  - Infographics/posters.
  → Bearing in mind illiteracy rates
  - Concerts to spread message and ↑ engagement.
- Experience sharing
  - Patient peer education.

❖ Wide range inclusion accommodating younger women/adolescents.

- Research teams should do community engagement.

EDUCATION

PROGRAMME

- **Community**
  - Informal teaching
  - Peer to peer education
  - Involving religious and traditional beliefs.
  - Involving partners, family.
  → Monthly workshops.
  → Targeting pregnant women.
  → Invite other women in community.
  → Delivered by in charge & MCA & Community health workers.
  → Get support from community leaders, leaders in health care.

- **Health facilities**
  - ANC
  - → Signs & symptoms
  - → When to seek help
  - → Complications involved
  - → Preventative measures. i.e regular care & BP checks.
  - One to one with.

Patient & care prouder.

- ❖ In waiting room easy to understand, info graphic/poster admission to hospital.
  - → Those diagnosed explained condition, treatment (side effects), procedures-i.e termination, early delivery.
  - One to one health care provider.
  - Follow up with “admission counseling”
  - → Chunk & check.
  - → Training well in care workers

Group -3

INTERVENTIONS FOR PRE-ECLAMPSIA MANAGEMENT

POLITICAL

FINANCE

GOVERNANCE & LEADERSHIP

- POLICY
- **SERVICE DELIVERY**

DRUG DELIVERY

- TECHNOLOGY
- COMMODITES & EQUIPMENT

DRUGS EXPIRING DUE TO IMPROPER/LACK OF PROCEDURE.

Government has increased health commodities funding from 0% (fully donor funded) to 50%.

Introducing Sanctions for not Adhering to policy including communities in determination of policies that affect health ⟶ increase sense of ownership

IMPROVING
SERVICE DELIVERY AT THE POINT OF CARE

Clearly define governance structure for –pre-eclampsia/eclampsia management.

Case of PE comes to facility.

- Power struggle between midwife & CHO
- Midwife and doctor.

PRE-ECCAMPSIA

- Delays Good
- Care
- Bottlenecks to quick care

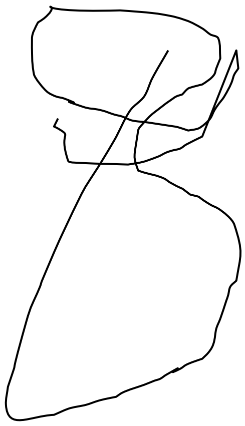

Midwife or MCHA
Sent to be examined
CHO

PATIENT

Who is Accountable?

RHFP Programme Management

- Start from facility/ward management level.

SOLUTION: TRIANGE AND SEND TO ECLAMPTIC WARD

RED LIGHT STATE DOSE & SEND. DON'T WAIT

Group -4:

Interventions for Charge-Cultural/Social

- Public Health Education Campaign:
  - ✓ Involve communities in each district to develop by managers for campaign & address….myths around eclampsia through a locally tailored approach.
  - ✓ USE Agents of change (women that had eclampsia in the past) to be advocates & spread awareness (slow /patient with good outcomes & I patient case with mobility/bad outcomes).

- **Religious Leaders Engagement & Training**
  - ✓ Engage & train TOT of in leaders cross-country (esp in rural area).
- Tie key messages on PET into ANC health talks & Patient health awareness.
- Work with Education Partners to integrate PET (e.g Manager into:

- Schoool Ecslicalia
- School clubs

- University –secondary school curriculum

- Train & Engage traditional Bundu Society leaders to spread awareness on Eclampsia & its management.

- **PUBLIC HEALTH EDUCATION**
  - ✓ Involve communities in each district to develop by manages on campaign & address myths around eclampsia.
  - ✓ Radio campaign: Religious stakeholders spread the word; social media campaigns, health talks.
  - ✓ Use Agents of change (women that had "lampsia in the past) to be Advocates & spread awareness show a patient with a good outcomes & I patient with bad outcome.

STAKEHOLDERS ENGAGEMENT

- ✓ Religious leaders are brought on board, trained & engaged to plakin Eclamppsia.
- ✓ Young women in schools, University ___ Secondary School curriculum.
- ✓ Women & the wides public via social media campaign.

- Religious Leaders Engagement & Training
  - ✓ Engage & train____ TOT of Religious leaders cross-country (up in rural HZR Communities).
  - ✓ Follow up: QR meetings.

Work with Education partners to integrate Eclampsia in unto school intervention & school clubs.

Work with FGM campaigns to address Bondo Society.

Group -5

Interventions for Change (Economic)

COSTS

Pre-hospital

Loss of income-if woman admitted

NEMS

Transport < Personally funded

In hospital

Food

Healthcare- “free” healthcare is not free-it’s not working no accountability----- people take advantage+ charge/equipment/drugs not available –have to buy it /blood-250,000 per unit.

INTERVENTIONS

1. National health insurance-contributes + have a card.
2. Birth preparation –saving, _____ EDUCATINO (CHW), knowledge mobilization from CHW.
3. Service charts-outlining what is free & what you pay for.
4. Maternity insurance scheme-covers basic package.
5. Blood donation scheme-at 34/40 bring 2 relatives to donate blood.

6. Working in Ghana, husband/head of household pays at start of pregnancy, people need to have confidence in it.
7. Community transport-all contribute in advance.
8. Cost recovery.
9. Sustainability of CRADLE- EDUCATION AROUND REPAIRING-80% CAN BE FIXED.

① Facilitators +

Solutions:

Data = Story

community sensitisation

traditional leaders involvement. → champions.

SMAGs [Safe motherhood action groups]

→ high risk registers.

→ Pictures → curriculum

~~District health~~ office

supplies

strengthen peripheral [illegible].

MDSR - implement action plans

champion districts.

Sensitise workforce.

Sharing true stories.

• Add Planned Del to SMAG curriculum

Peer support

outreach.

Barriers −

women's voices not often heard

scared of IOL → pain

→ ↑ contractions

↳ spread of fear/misinformation

cultural beliefs;

suspicious of early labour

- husband; MiL

- infedility

Cost.

Fear of NICU

childcare.

Fear of C/S - "lazy"

Family influence - consent.

BP machines / urine dips.

The system - Quality

Grandmothers

Infrastructure

CS CamScanner

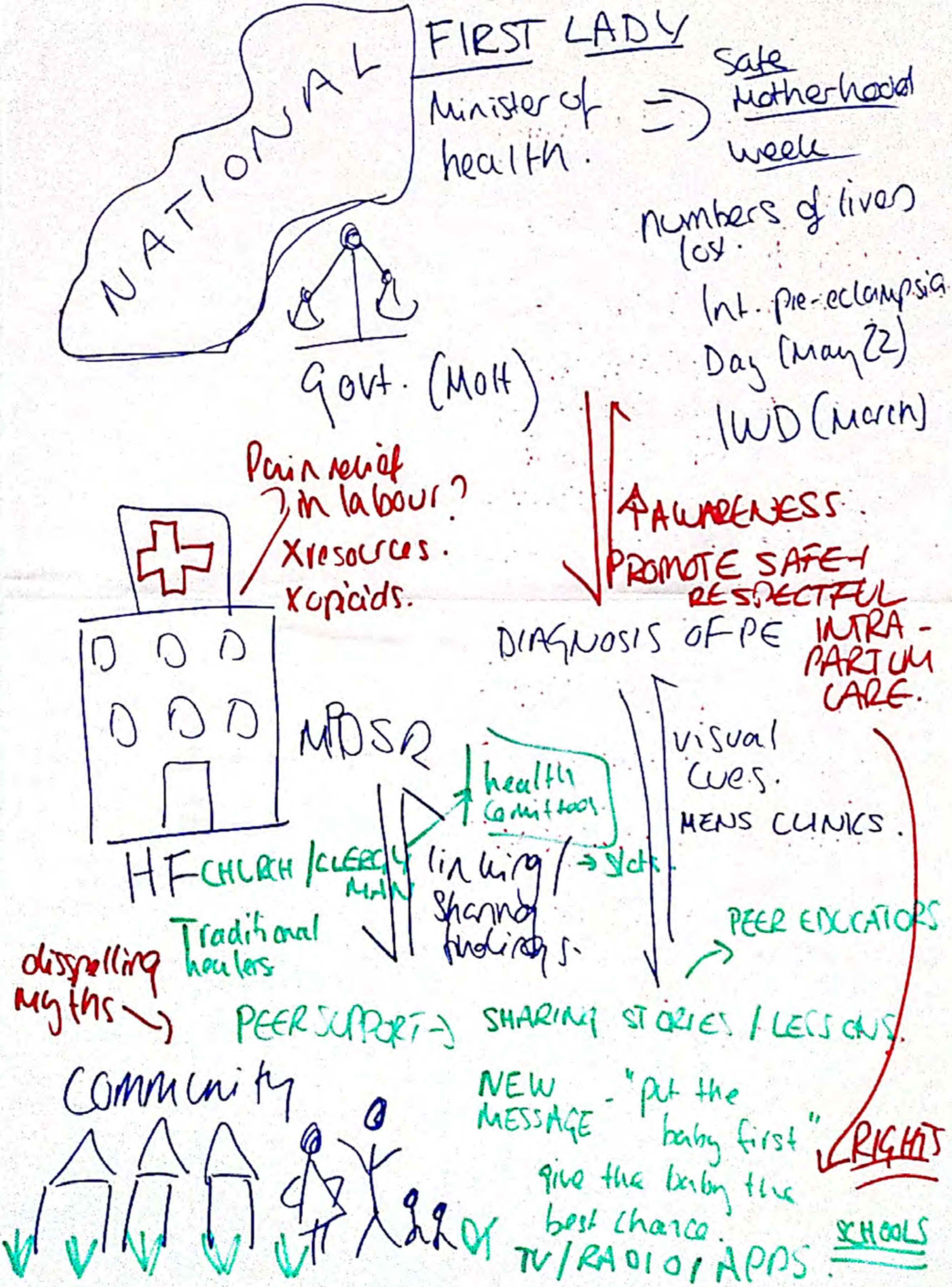

CS CamScanner

⑥ Stigma towards

Pr.
Attitudes & Beliefs around planned early delivery.

① Induction of labour (forced labour) generally perceived to be painful

② Fear of delivery by c/s risk

③ Fear of having preterm baby who will end up in NICU

④ Fear of outcome of the Process

⑤ Decision making at family and community level.

CamScanner

⑥ Stigma towards a woman Perceived to have 'failed' to carry a pregnancy to term.

⑦ Desire for natural birth

CS CamScanner

Feasibility of planned early delivery.

Facilitators	Barriers
- Generally acceptable expected fetal weight after 34 weeks GA.	- Referrals with logistics and cost implications
- Early delivery after 34 weeks already practiced for known common conditions	- Access to facilities which can offer induction ± C/S.
- CRADLE 4 having been conducted in Malz including our setting	- Uncertain gestation age
	- Lack of knowledge about new evidence
	- Fear of referrals among patients

Facilitators	Barriers
- Contribution of Pre-eclampsia to Maternal Mortality	- Lack of capacity in our NICU.
- Faith/trust in the health-care workers.	

Creating feasibility and acceptability

Community

1) Integrate planned early delivery for pre-eclampsia into the curriculum for SMAGs / CHAs

2) Neighbourhood Health committees can be channels of communication to community leaders

3) Community outreach programs to engage community members to update them on early delivery for late preterm eclampsia
- Clinicians / public health specialists

CamScanner

- use different media to deliver evidence based materials

(4)

Clinicians

(1) Update guidelines on management of Pre-eclampsia - UNZA obs/gyn guidelines due for review (opportunity window).

(2) Teaching Institutions to include in their curriculum new evidence-based knowledge

(3) Presentation during Safe motherhood Technical working committees.

(3) Dissemination through professional association meetings - MAZ | ZAMARACO. RDAZ

(4) Mentorship tools

- Pregnant ♀ fear planned early delivery.
- NICU - pregnant ♀ fear this. + the baby being under light in an incubater.
- 'Empower the provider' to give the right 'package of information' to the ♀'
  if the HCW doesnt believe they can't explain/counsel ♀
- * PARTNER involvement influence* ♀ decision - increase awareness. fines
  (general family members very * influential) * influential community member involvement*
- monitoring issue/staffing providing quality care + close monitoring to planned deliveries. (±OL)
  → failed IOL ↓ Lower level hospital ↓ needing to refer ♀ to higher level facilities.
- NICU facilities are limited.
- convincing clinicians - to move away from the old guidelines.

CamScanner

HEALTHCARE System / Healthworker PERSPECTIVE :-

- Empower the healthworker to counsel ♀
- concerns :- are we increasing referrals to support planned early delivery?
- Are we ↑ monitoring needed to support I.O.L planned early delivery?
- Are we ↑ use of Limited NICU facilities?
- cost/ benefit value ??

WOMEN :- Early baby will go under light in incubator which is bad. And baby will grow up with problems.

- engaging ♀ families, partners, influential family members + community members to support ♀ to have planned early delivery.

CamScanner

Community (♀ + families) ↗ Gatekeepers ↗ 'BIG BOSS'

- × sensitisation: communicating with traditional leaders + groups. + RELIGIOUS
- × social media / Radio Broadcasts / TV
- × SMAGS: Bridge between community + hospital: 80% in community + 20% in hospital ↳ NHCS
- × civic leaders 'MODERN CHIEFS' - elected by community.
- × EDUCATION for children so that anything other than 'normal delivery' is not stigmatised.
- × Example from Sierra Leone: community film screening (↱ in A.N.C. → FILMS AND EDUCATION) to target all ages + all types of community member.
- × incentives for male partners to come to Antenatal care.

CLINICIANS:

- × training + orientations. → QUs + issues need to be answered + addressed. 'clearly packaged'.
- × Clinicians 'Living what they say'.
- × New guidelines. - UPDATED.
- × TEACHERS. NURSING COUNCIL / HPC-Z / ZPA REGULATION of CURRICULUMS.
- × Knowing the numbers - Does it truly translate?

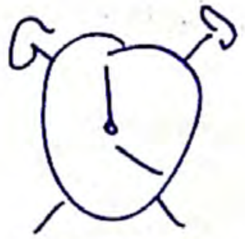

PROGRAMME managers + policy makers. £ $ ZMW

- seeing evidence put into practice in real life
- creating policy documents.
- Bottom up approach. - taking evidence to the top MOH, ADMINISTRATOR. SAVES MONEY cost effectiveness

CS CamScanner

x showing evidence that P.E.D ↓ costs ass:

- severe pre-eclampsia complications e.g dialysis.
- Long hospital stay.
- Prolonged NICU admission.

CamScanner

Current attitudes + beliefs

Facilitators

Previous experience.

Peer influence.

Education — right education is key to universal implementation

→ Novel ways of educating due to poor literacy.

Trust in doctors/midwives, especially *senior doctors — junior docs may spend 2-3 days persuading women, senior doctor convince women in 2-3 mins.
- First contact is key.

Healthcare workers feel it is feasible to rollout.

*All speaking same language - junior + senior, dr + MW.

Planned early delivery did not ↑CS in CRADLE-4 (↓CS in PHOENIX - UK)

Social media - women sharing personal experience of PE (both facilitator + method of implementing).

APEC - Zambia

→ best is messaging from someone you trust within the community.
more reliable than TV/radio.

Honesty + integrity in counselling - planned early delivery → "more likely to save your baby", not "it will save your baby".

Barriers

If your baby goes to SCBU he/she will never make it.

*Early delivery → "your baby will be too small"
→ "fear of immature baby; how long will I be in hospital?"

People don't know what PE is / what complications.

If woman comes alone, need to tell mother-in-law, husband, biological parents - may be out of town.

*No symptoms to PE - associate disease with feeling something, whereas lack of symptoms harder to believe / act on, "I'm not feeling it"

*Joint decision making with whole community
- "your pregnancy, but whole family/community's child"

Women may refuse treatment/delivery, even if previous PE.

*Cultural beliefs - PE results from eg. unfaithfulness in marriage, other bad behaviours, fear that seizures are contagious, especially in rural areas.
- if need CS - man has been unfaithful.

→ Noone wants to take responsibility of making decision to deliver.

"Pre-eclampsia" not in local language.

"My BP has always been (N)"

"Forced labour" - not natural
*more painful

Not considered a strong woman if you have CS. 2 CS - can be end of marriage. IOL better than CS.

CS taboo

CamScanner

How feasible / acceptable among different stakeholders?

<u>Women + Families</u>

- Add to current AN education - specific to PE - add PE symptoms to danger signs, + add planned early delivery >34/40
  [Pre-conception care is very poor]
- IEC educators need training / education
- ↳ taking IEC education out into the community (because "community pregnancy")
  e.g. by radio, in church (not schools)
  TV / soap opera - woman with PE built into plot
- Need to achieve education + waking women up, without scaring them - dead babies are a daily reality
- APEC - Zam to run study days - to count for CPD
- Dissemination of information depends on how well women understand it - rural / vs. urban, rural linkage to urban areas

<u>Healthcare Workers</u>

- PE is in curriculum for MW / doctors,
  ↳ include planned early delivery from 34/40
- Influence post-graduate guidelines.
- key to include paeds / NICU staff,
  have to emphasise that NICU use (+ other outcomes) is not increased by planned early delivery.
  eg. neonatal conferences, meetings etc.
  <u>NB</u> 1° reason for admission to NICU = prematurity
- ↑ KMC capacity.
- 22/5 - PE Day!
