# Supplementary material for: A novel approach to expedite evidence to impact in pre-eclampsia: co-developed policy labs in Zambia and Sierra Leone
Source: BMC Glob Public Health. 2025 Jan 7;3:3. doi: 10.1186/s44263-024-00116-8 (PMC11707905; doi:10.1186/s44263-024-00116-8)
Supplement: Supplementary file 4 — Additional file 4. Raw data: ethnographic observation notes [file 44263_2024_116_MOESM4_ESM.pdf]

Policy Lab workshop on the 7 march 2023 time 09:30

The workshop was held at the western part of Freetown, which is a settlement of the creole that were freed from slaves during the slavery era. It has a big river, which is used to cross to go to the northern part of Sierra Leone where the airport is located. It has a market, church, military barracks, and some factory. Its also have the oldest school in Sierra Leone which is the grammar school. It is also have a graveyard that is used by both the Christian and Muslim. The workshop was held at the main street opposite the church and the market. It is a big compound with 3-conference room, and a lounge which has a long table and chairs which is used for lunch and breakfast and another sets of couch with a table at the Centre and there are some books which most of us look at. On the gate, there is the policy banner, which is easily recognised by the invitees. Its painted grey colour and its seems so good and everyone like the place was happy to know about it because it is the first time for most of use to go there. We used the 3 conference rooms for the workshop for the different team to discuss the cradle and the ways to make it known by the communities at large.

35 people attended the workshop 17 female, 18 male. Three representative from Zambia, professors, DMOS, DHS sisters, religious leaders, matrons, DR, traditional healers. The meeting was delayed because of the compatibility of the computer to the tv for presentation. The meeting was started by 09:52 with an opening individual prayers and introductions from the participant their names and position and the organization. The Zambia first did her presentation of their own policy lab that they held few weeks ago and their success, failure, and they are here to look the similarity and difference between us. The coordinator did a brief talk on the schedule for the day and the importance of the policy lab

A female facilitator, who is a former matron and is part of the cradle team, held the sections. She was able to take control of the presentation and did her work perfectly despite sometimes she seems to cut people off not to exceed the limited time. It was a very interactive workshop where everybody has his or her thought on how to make the people know about the cradle device and its importance.   It was a very interactive session by all and incorporating everyone's ideas. There were different groups up to 5 with each where there is a facilitator and it was not the same amount of persons and its varies and not gender balance and not by profession it was randomly selected by the coordinator. The first group thee were comprises of 5 people, 3 female 2 male, 1 professor,1 Dmo,and 3 matron 2nd group comprises of 6, 3 female and 3 male 3rd group 5 2 female 3 male. Fourth group 7 1 female 7 male 4 DR, 1 traditional healer,1 statician,1 matron the 5th group 5 6 2 female 4 male. The most interesting group was group 3 where they have one district health officer who knows the health system way too much and knows the lapses, the bad, the challenges, the power dynamic within the health system, the bad attitude of health workers not communicating with patient, the problem of heirachycha in the the phu between midwifes and cho and the no complain of patients system the advantages healthworkes uses on patient and she has enough to say but because of time she was stooped and when she was talking everybody was saying its true and that she explains exactly what is happening and everyone was supporting her and there were momentum of laughter and fun everyone was clapping doe for hitting the nail on the head. The different groups did their presentation from the 15 min sessions they had. Most of the disccution were want they know and what they are lacking in the hospitals and phus and many options arises but because of limited time only few where discussed. Like lack of trust, low salary, no standard equipment, lack of refrshers training, power dynamic, lack of drugs, lack of communication, bad terrain, lack of emergencies equipment, delays, attitude of health workers not involving stakehoders and community who are using the services, not using local names so the users can understand the services they are receiving more. Professor gives a brief history about the cradle machine and how it

was developed and how its cam about and the successes it has achieved so far where they have been used. Everyone was concentrating during the Professor presentation despite some were playing with their phones, some taking photos of the presentation. No question were allowed for professor because of time but he said he is available for any question during tea break. I notice some people were having chat with him during tea break and the break lasted for 30 min and all were called to meet at the and have another discussion before going to the small sessions. The coordinator explains what to done in the next sessions and showing each team a piece of paper and the written on it and the discussion should be based on the topic she provided. The team also goes to the smaller rooms as before and started with sessions. This session was for 15 mins and all were called to do their presentation on the ways or channel to which the pre-esclapsia and esclapsia will be known by all in the country expect to do 5 min presentation and not to mention what the other group has already said. So all 5 the groups presented and they mention the ways by posters, billboard, radio program social media, sensitization in schools, it shoud be added in the school syllabus ;, TBA, nurses during outreach and antenetal mosques. Churches, ataya base and the involvements of the stakeholders in the community, drama and to use local langugaes that the native people will understand and its was so interested and everyone was alert to listen. By this time, I notice some DMO have already left and someone comes in after lunch break. During lunch brawl we had snacks and tea and its was so delicious but some are murmuring that it was too small and that you are only getting it once so I smiled and said we don't you to get fat and they laught. There was a very interesting episode that happen between the facilitator and the traditional healer, where the facilitator called the TBA and the traditional healer said they should be called traditional healers because they are under his supervision ;and that they should they the tradtitonal healer are doing a wonder job to promote health in whatever way and the facilitator seems to be annoyed a little and tries to tell the him that we called TBA and that how its remain we don't called traditional healers because we know the difference between traditional healers and TBA and he said no they must called tradtonal healers and he seems to proud of his job and all started saying okay we know they are under your umbrella but we are differencing it for us to get a better understanding, and the facilitator then calm down and said okay I know your job is important and we respect that and we like to proceed to the next presentation and everyone was laughing . during lunch time we had options to the food we want that is rice and beans or fishes and vegetables and most of the people were saying the food is small the one that they served and the couscous had no stew and its dry. But the ones that orders the beans and rice were saying the rice is small.

There was a short film which is about Pre-eclapsia and eclapsia which captured the attention of all and everyone was paying attention and the movies moved all the people especially the DR and Matrons they were acting as if it's a reality and they praying for the patient not to die or the child too blaiming the motherlaw for not accepting her daughter inlaw to go the clinic a nd happy for the daughter inlaw friend who persisted for her friend to be taken to the clinic and both the mother and the child were saved. So one DR was saying to use that kind of medium should be use to pass the message the to the community people. Professor did another 5 mins talk through and the importance of all to be involve and give their own take so that the policy lab will be of great help to us all and also said goodby to a colleague for she will be leaving the country and the project and will be going forward and thank her for her great work and she was waving her hands and the matron were saying "u dae go lef we" meaning are you leaving us and she smiles and said yes but who knows I will be back another time. The facilitator thank each one for participating and the importance for the policy lab and the involvement of all and coordinator thank us all. We were told to go for a drink and snacks which is 5 mins drive walk and all went there and there a lot of different snacks, and we were acssesing it by giving it our local food names for resemble the typical food we

have like one that were served seem to be like our cassava bread same size and stew inside and one like our voray which we normally use to name our child during the naming ceremony but everyone seems happy and tastes all the different types of snacks.
